# Anti-Periodontitis Effects of *Dendropanax morbiferus* H.Lév Leaf Extract on Ligature-Induced Periodontitis in Rats

**DOI:** 10.3390/molecules28020849

**Published:** 2023-01-14

**Authors:** Ye jin Yang, Jun-Ho Song, Ju-Hye Yang, Min Jung Kim, Kwang Youn Kim, Jin-Kyoung Kim, Yeung Bea Jin, Woo Hyun Kim, Suk Kim, Ki-Rim Kim, Kwang Il Park, Hu-Jang Lee

**Affiliations:** 1 College of Veterinary Medicine, Gyeongsang National University, Jinju 52828, Republic of Korea; 2 Korean Medicine (KM) Application Center, Korea Institute of Oriental Medicine, Daegu 41062, Republic of Korea; 3 Department of Dental Hygiene, Daegu Health College, Daegu 41453, Republic of Korea; 4 Department of Dental Hygiene, Kyungpook National University, Sangju 37224, Republic of Korea

**Keywords:** *Dendropanax morbiferus* H.Lév, periodontitis, anti-inflammation, anti-oxidant, alveolar bone, ligature-induced periodontitis

## Abstract

Periodontitis is caused by pathogens in the oral cavity. It is a chronic infectious disease that causes symptoms including gingival bleeding and tooth loss resulting from the destruction of periodontal tissues coupled with inflammation. *Dendropanax morbiferus* H.Lév (DM) is a natural product that exhibits various biological activities with few side effects. In this study, the potential of DM leaf hot-water extracts (DMWE) as a treatment for periodontitis was determined and its anti-oxidant and anti-inflammatory effects were evaluated. Compounds in DMWE were identified by high-performance liquid chromatography (HPLC) and nitric oxide (NO) and prostaglandin E_2_ (PGE_2_) production was measured in RAW 264.7 cells. We measured the gingival index and gingival sulcus depth, and micro-CT was performed in vivo using a ligature-induced periodontitis rat model, which is similar to human periodontitis. The DMWE-treated group exhibited a decrease in cytokine concentration and relieved the gingival index and gingival sulcus depth compared with the periodontitis-induced control group. In addition, micro-CT and histological analysis revealed that DMWE exhibited anti-inflammatory effects and improved alveolar bone loss in periodontitis-induced rats. These findings suggest that DMWE has excellent anti-oxidant and anti-inflammatory effects that protect and prevent periodontal tissue damage and tooth loss caused by the inflammatory response.

## 1. Introduction

Periodontal disease is a group of diseases affecting periodontal tissue and includes gingivitis and periodontitis [1]. The inflammatory process begins in the gingiva and proceeds to gingivitis. Gingivitis is a common oral disease that is caused by a bacterial dental polymicrobial biofilm. If left untreated, the gum inflammation accelerates to periodontitis, an irreversible and destructive autoimmune response [2]. Periodontitis damages the surrounding tissues and the supporting alveolar bone [3], resulting in the loss of collagen fibers and attachment to the cementum surface, as well as deeper periodontal pockets and alveolar bone loss [4], which eventually leads to tooth impaired function and tooth loss due to bone damage [5]. Approximately 11.2% of the world’s population are affected, resulting in decreased quality of life and economic pressure because of dental care costs [6].

Periodontitis is a prevalent oral disease characterized by chronic inflammation. It is caused by an immune response against various types of Gram-negative anaerobic bacteria [7]. Lipopolysaccharide (LPS), the major component of the outer membrane of gram-negative bacteria, induces the production of inflammatory cytokines, such as tumor necrosis factor-alpha (TNF-α), interferon-gamma (IFN-γ), interleukin-1beta (IL-1β), and interleukin-6 (IL-6). Subsequently, it infiltrates connective tissue and eventually induces localized inflammatory responses [8,9], which leads to clinical attachment loss and bone destruction [10]. Periodontitis causes adult onset and necrotizing forms at early onset and is graded based on the severity of disease and the rate of progression [11].

Nitric oxide (NO) and prostaglandins (PGs) are important mediators of inflammatory responses [12]. Cytokines, prostaglandin E_2_ (PGE_2_), lysosomal enzymes, and free radicals have been implicated in inflammatory reactions. In the macrophage inflammatory response, the expression of infectious cytokines, such as TNF-α, is induced, and inflammation induces inducible nitric oxide synthase (iNOS) and cyclooxygenase-2 (COX-2) genes to produce NO and PGE_2_ factors [13]. When inflammatory mediators including iNOS, COX-2, TNF-α, and IL-6 are activated, NO is overexpressed, which results in vascular enlargement, tissue damage from the inflammatory response, mutagenesis, and damage to nerve tissue [14]. In addition, overexpression of COX-2 promotes tumor cell resistance to invasion and apoptosis through the overproduction of PGE_2_ [15].

*Dendropanax morbiferus* H.Lév (DM) leaf (DML) extracts were reported to possess anti-inflammatory and anti-oxidant activities by modulating apoptosis in various human tumor cell lines and osteocarcinoma cells [16]. The bioactive components in herbs and plants can prevent and treat many diseases. Because of its ability to cure various diseases and maintain health with little to no adverse effects, there has been increased interest in the extraction and separation of a wide range of bioactive compounds from herbs and plants [17,18]. DM is known as a highly physiologically active material [19]. It is an endemic, evergreen plant from Southern Korea [20] and has been used in traditional medicines in Asia [19], because it exhibits anti-oxidant [21], anti-diabetic [21,22], and hepato-protective [23] effects. Previous studies have indicated that DML hot-water extracts (DMWE) have increased anti-oxidant enzyme activity and exert protective effects against diabetes, cancer, atherosclerosis, and kidney toxicity [19,21].

In this study, we evaluated the anti-oxidant and anti-inflammatory effects of DMWE, its effects on alveolar bone loss, and amelioration of periodontal inflammation in a periodontitis-induced rat model.

## 2. Results

### 2.1. Analysis of DMWE by High-Performance Liquid Chromatography (HPLC)

**Figure 1:**
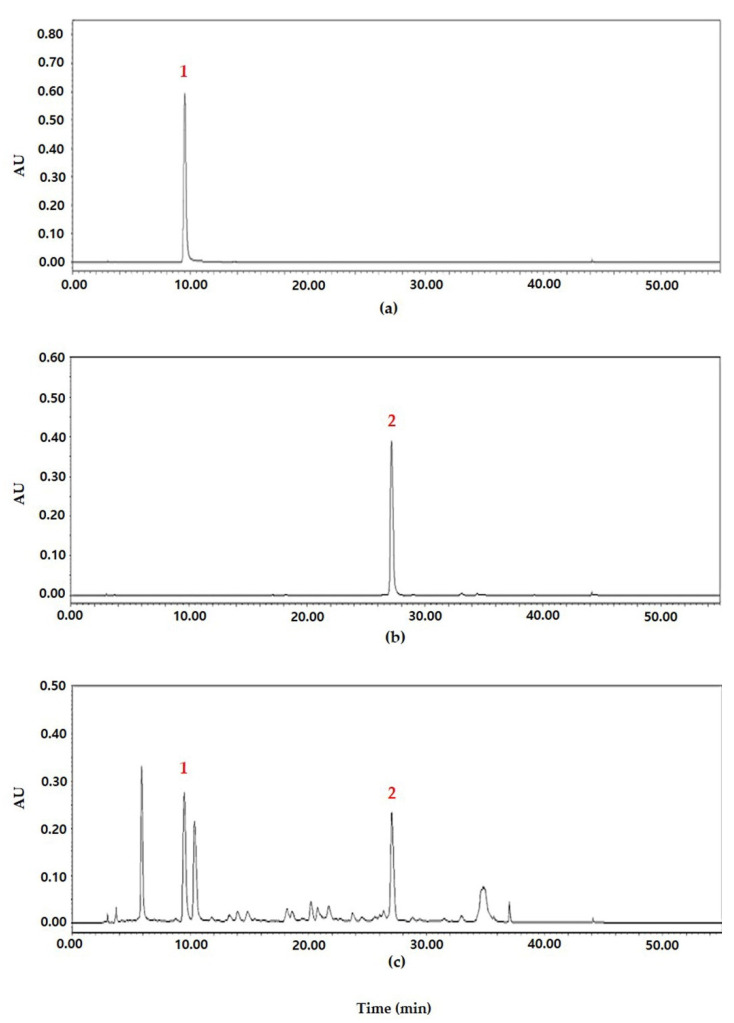
HPLC chromatogram of flavonol compounds in DMWE. (**a**) Chlorogenic acid standard solution, (**b**) rutin standard solution, (**c**) DMWE sample solution. Chlorogenic acid (1), rutin (2).

Chlorogenic acid and rutin standards, which are the active ingredients of DMWE, were used to establish HPLC analysis conditions. Two flavonoids were identified according to peaks obtained by HPLC chromatography at a wavelength of 310 nm. Two flavonoids were identified as chlorogenic acid (1) and rutin (2) at concentrations of 2.94 ± 0.15 mg/g and 7.81 ± 0.40 mg/g, respectively (Figure 1).

### 2.2. Total Polyphenol Contents of DMWE

To characterize the compounds in DMWE, we measured the total polyphenol and flavonoid content. The total amounts of polyphenol and flavonoid were 55.32 ± 2.15 GAE mg/g and 11.98 ± 0.12 QC mg/g, respectively (Table 1).

### 2.3. Anti-Oxidant Effects of DMWE

**Figure 2:**
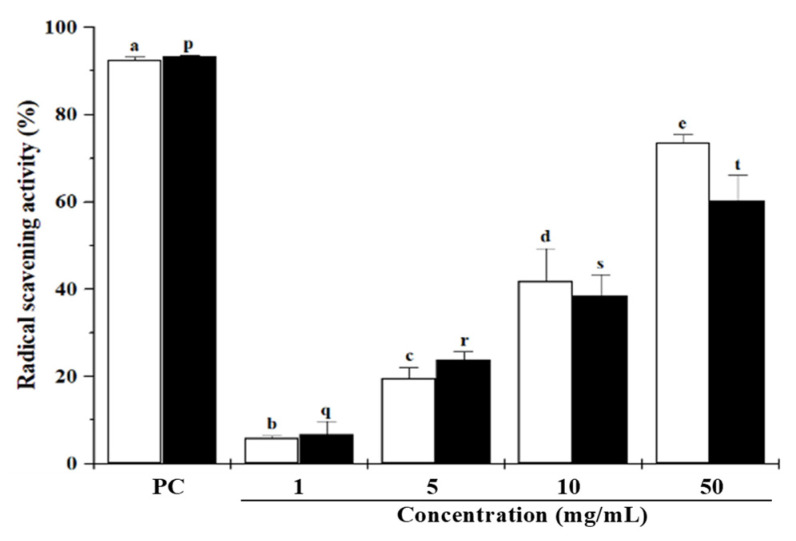
DPPH (□) and ABTS (■) radical scavenging activities of DMWE. Positive control, 1.0 mg/mL of vitamin C. Different superscript letters (a, b, c, d, e) indicate statistical significance in DPPH radical scavenging activity (*p* < 0.05). Different superscript letters (p, q, r, s, t) indicate statistical significance in ABTS radical scavenging activity (*p* < 0.05).

To evaluate the anti-oxidant effects of DMWE, we measured the DPPH and ABTS radical scavenging activity. DPPH and ABTS radical scavenging activity were increased in a dose-dependent manner. Vitamin C was used as a positive control (Figure 2).

### 2.4. Anti-Inflammatory Effects of DMWE

**Figure 3:**
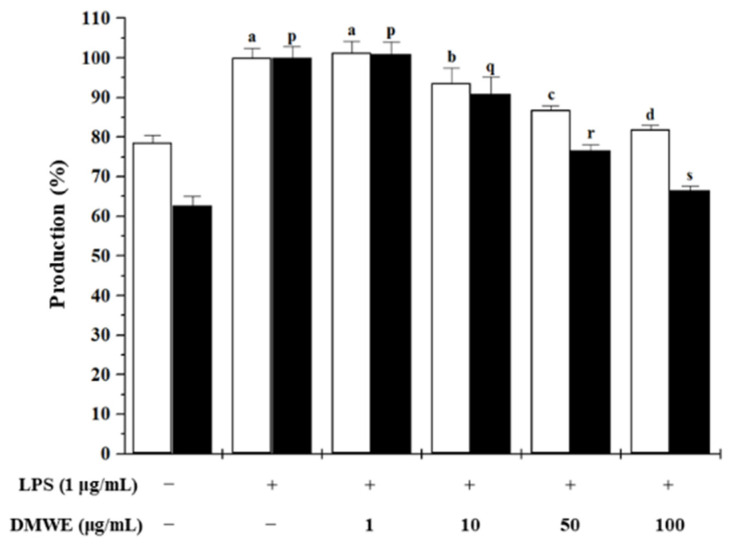
Effects of DMWE on LPS-induced NO (□) and PGE_2_ (■) production in RAW 264.7 cells. LPS, liposaccharide; DMWE, hot-water extracts of DML. Different superscript letters (a, b, c, d) indicate statistical significance in nitric oxide (NO) production (*p* < 0.05). Different superscript letters (p, q, r, s) indicate statistical significance in prostaglandin E_2_ (PGE_2_) production (*p* < 0.05).

NO and PGE_2_ are markers of inflammation caused by periodontitis in rats. We measured NO and PGE_2_ levels to determinate the inhibition of inflammation activity. Both NO and PGE_2_ were decreased and dependent on DMWE concentration (Figure 3).

### 2.5. Body Weight

To determine whether there was a change in body weight caused by periodontitis in rats, body weight was measured before and 1, 2, and 3 weeks after DMWE treatment. There was no statistically significant difference in body weight for all groups before or after 1, 2, and 3 weeks DMWE treatment; however, all groups exhibited a tendency toward increased body weight in a time-dependent manner. Following DMWE administration for 3 weeks, the weight gain of DMWE-2 × MBC was statistically significantly increased compared with the PC (*p* < 0.05), but there was no significant difference in weight gain for all DMWE-treated groups (Table 2).

### 2.6. Analysis of Inflammatory Cytokines in Serum

We measured the concentrations of the inflammatory cytokines, TNF-α, and IL-6, in the serum of periodontitis-induced rats. TNF-α and IL-6 levels were decreased in a dose-dependent manner in the DMWE-treated groups. TNF-α and IL-6 concentration decreased compared with the positive control group (PC) (*p* < 0.05) in DMWE-MBC and DMWE-2 × MBC, whereas TNF-α concentrations in DMWE-2 × MBC were decreased significantly compared with the DMWE-MIC group (*p* < 0.05) (Table 3).

### 2.7. Measurement of Gingival Index (GI) and Gingival Sulcus Depth

To determine the degree of gingival relief following the administration of DMWE to periodontitis-induced rats, the GI and gingival sulcus depth were assessed. The GI and gingival sulcus depth were decreased and dependent on the concentration of DMWE in the DMWE-treated groups. DMWE-2 × MBC exhibited a significantly decreased GI compared with the PC (*p* < 0.05), and the gingival sulcus depth was significantly decreased in DMWE-2 × MBC compared with the PC (*p* < 0.05), but was significantly higher compared with the normal control group (NC) (Table 4).

### 2.8. Radiographic and Microcomputed Tomography (CT) Observations

**Figure 4:**
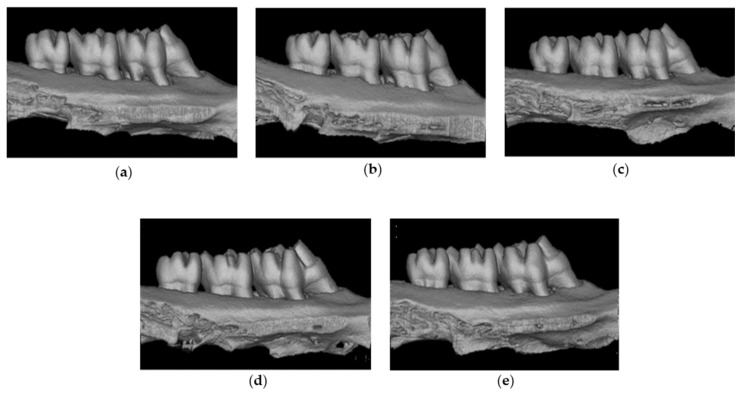
Effect of DMWE on alveolar bone loss and tissue damage. The images of bone surrounding the molars were analyzed by three-dimensional microcomputed tomography and are shown here to represent each group: (**a**) non-ligature-control, (**b**) ligature-control, (**c**) ligature-DMWE-MIC administered DMWE 0.313 mg/rat, (**d**) ligature-DMWE-MBC administered DMWE 0.625 mg/rat, and (**e**) ligature-DMWE-2 × MBC administered DMWE 1.25 mg/rat.

To determine the loss of alveolar bone resulting from periodontitis in rats, we conducted micro-CT imaging on each group (Figure 4). In the PC, loss of alveolar bone resulting from periodontitis was clearly observed and the alveolar bone height was lowered to the furcation area (between the root and root) for some teeth (Figure 4b). In contrast, compared with the PC, the amount of alveolar bone loss was decreased in the DMWE-MIC and DMWE-MBC groups. In addition, progression of periodontitis was relatively slow in DMWE-MIC, few inflammatory indicators were observed around the apical end, and bone density was slightly decreased in the DMWE-MBC group (Figure 4c,d). In the DMWE-2 × MBC group, alveolar bone loss was significantly reduced compared with the PC and there were almost no signs of inflammation around the apex and a decrease in bone density (Figure 4e).

### 2.9. Alveolar Bone Resorption Evaluation

**Figure 5:**
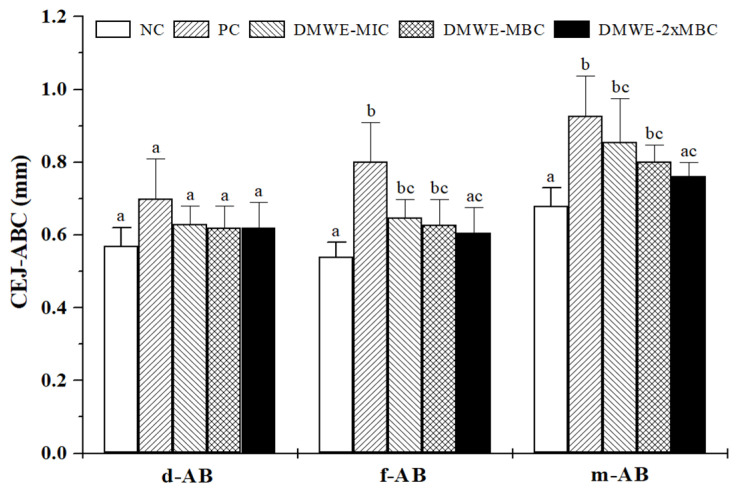
Linear measurements to assess alveolar bone loss from the cementoenamel junction (CEJ) to the alveolar bone crest (ABC) (CEJ-ABC) of the maxillary second molar by micro-CT section analysis. d-AB, the distance from the CEJ to the ABC on the distal side of the molar; f-AB, the distance of the CEJ to the ABC on the distal side of the mesial root at the furcation of the molar; m-AB, the distance from the CEJ to the ABC on the mesial side of the molar. NC, normal control; PC, positive control treated with distilled water; DMWE-MIC treated with 0.1 mL MIC (3.13 mg/mL) of DML hot-water extracts (DMWE); DMWE-MBC treated with 0.1 mL MBC (6.25 mg/mL) of DMWE; DMWE-2 × MBC administered with 0.1 mL 2 × MBC (12.5 mg/mL) of DMWE. ^a,b,c^ Mean values with different superscript letters in the same column indicate statistical significance at *p* < 0.05 by one-way ANOVA.

The distance between the coronal plane of the cementoenamel junction (CEJ) and the alveolar bone crest (ABC) was measured to evaluate alveolar bone resorption. All DMWE-treated groups were decreased (d-AB) in a dose-dependent manner compared with PC, but not significantly. In the case of f-AB, DMWE-2 × MBC were significantly decreased compared with PC. Finally, m-AB was decreased in DMWE-2 × MBC and it was significantly decreased compared with PC (*p* < 0.05) (Figure 5).

### 2.10. Alveolar Bone Analysis Evaluation

The effects of DMWE on periodontitis were analyzed by measuring alveolar bone density and morphological microstructural parameters. Bone mineral density (BMD), bone volume (BV), tissue volume (TV), and BV/TV ratios tended to increase in a dose-dependent manner in all DMWE-treated groups. In particular, in DMWE-2 × MBC, the BMD, BV, TV, and BV/TV ratios were increased in a dose-dependent compared with the PC (*p* < 0.05) (Table 5).

### 2.11. Histological Analysis

**Figure 6:**
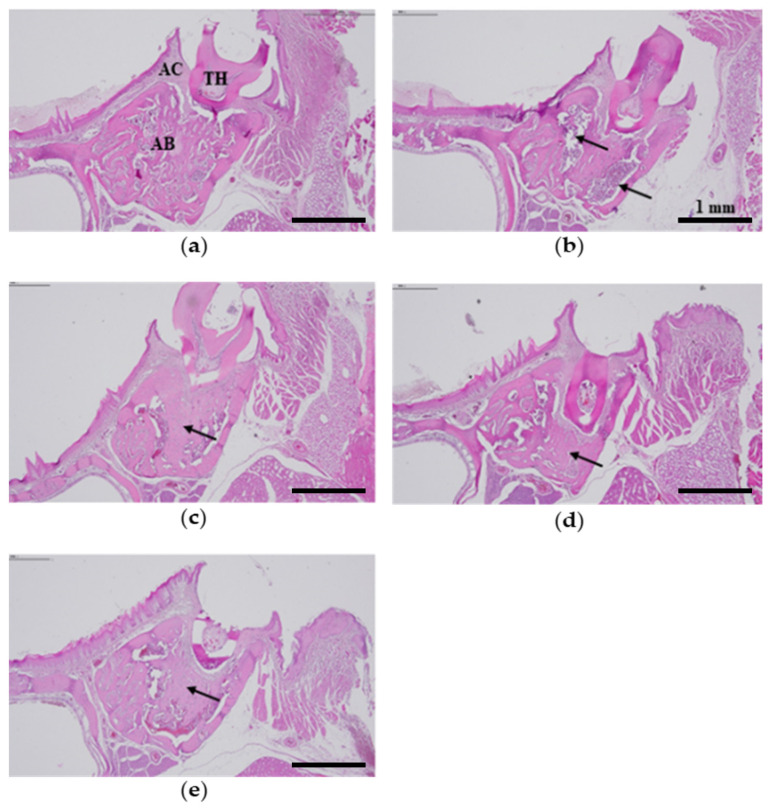
Representative H&E stained histological images of alveolar bone areas in rats with induced periodontitis after administration of DMWE for 3 weeks. The histological images shown are representative of each group: (**a**) non-ligature-control, (**b**) ligature-control, (**c**) DML hot-water extracts (DMWE)-MIC administered with DMWE 0.313 mg/rat, (**d**) DMWE-MBC administered with DMWE 0.625 mg/rat, and (**e**) DMWE-2 × MBC administered with DMWE 1.25 mg/rat. Magnification, ×40. Black arrow, alveolar bone density; AC, alveolar crest; TH, tooth; AB, alveolar bone. Scale bar = 1 mm.

The left alveolar bone in the rat maxilla was examined by H&E staining. In the PC, there was a significant decrease in periodontal bone density, but the inhibitory effects of alveolar bone loss were histologically observed in a dose-dependent manner in DMWE-administered groups. In particular, the BMD of alveolar bone in DMWE-2 × MBC was similar to that of the NC (Figure 6).

## 3. Discussion

In this study, DMWE was extracted with hot water to confirm the efficacy of DML on periodontitis. We identified the main compounds in crude extracts by HPLC responsible for the anti-oxidant and anti-inflammation effects in DMWE. Chlorogenic acid and rutin are polyphenol compounds belonging to the family of flavonoids associated with many anti-oxidant, anti-inflammatory, and anti-aging properties. Polyphenols and flavonoids, which are widely distributed in the plant world, are anti-oxidants that suppress the generation and action of reactive oxygen species. They are effective in preventing and delaying aging and preventing certain diseases. Flavonoids, one of the polyphenol species, are potent compounds with various pharmacological effects including anti-oxidant, anti-viral, anti-inflammatory, and anti-allergic activities [24,25,26]. DMWE contains a substantial amount of polyphenols and flavonoids (approximately 55.32 GAE mg/g and 11.98 QE mg/g, respectively). This suggests that DMWE has a high level of anti-oxidant activity (Table 1). As a result of confirming the anti-oxidant efficacy of DMWE using DPPH and ABTS, the radical scavenging activity was shown to be increased in a dose-dependent manner. In the highest concentration treatment group (DMWE 50 mg/mL), the radical scavenging ability of DPPH was 74% and that of ABTS was 61.3%. Based on these results, DMWE is considered to contain a high level of anti-oxidant activity (Figure 2).

Reactive oxygen species (ROS) can aid to kill invading harmful microorganisms, but when overactivated, it can become toxic to cells [27,28]. ROS cause intracellular damage to biomolecules and cell membranes [29]. An overabundance of ROS leads to increased oxidant load and reduced anti-oxidant capacity, resulting in oxidative stress within the affected tissues, leading to pathological changes [29,30]. In other words, a loss of teeth as their supporting structures become destroyed in periodontitis. In this study, it was confirmed that DMWE has an excellent anti-oxidant effect. Furthermore, its anti-oxidant effect can be expected to inhibit tooth loss because of periodontal inflammation.

Periodontitis is an inflammatory disease affecting oral health and is one of the humans’ most common chronic infections [31]. Periodontitis is an inflammatory disease that affects oral health and is one of the humans’ most common chronic infections. Clinically, the term for periodontitis stages is gingivitis-initial periodontitis-advanced periodontitis. However, most patients with periodontitis are a form of chronic inflammation of the periodontal tissue that progresses over a long period of time, becoming ‘chronic’ periodontitis [32]. Therefore, in this study, the periodontitis model induced by ligation for 3 weeks was designated as chronic periodontitis. Gram-negative *Porphyromonas gingivalis* (*P. gingivalis*) is a major pathogen of periodontal disease, and the disturbance of oral bacterial homeostasis induced by *P. gingivalis* infection plays an important role in the onset and progression of periodontitis [33]. LPS secreted by periodontal bacteria induces an immune response in macrophages and increases the expression of pro-inflammatory cytokines [34]. In our results, there was no change in cell viability up to DMWE 100 µg/mL, and production of NO and PGE_2_ in LPS-stimulated RAW 264.7 cells was decreased in a dose-dependent manner with DMWE treatment. When inflammatory responses occur, NO and PGE_2_ act as triggers to increase the degree of inflammation. PGE_2_ is a potent stimulator of the inflammatory response and bone resorption, and its production is associated with increased connective tissue in periodontitis lesions [35]. We also confirmed that DMWE inhibited the increased concentrations of TNF-α and IL-6 in the serum of periodontitis-induced rats in a dose-dependent manner. Our results are consistent with many studies that periodontal pathogens, including LPS from *P. gingivalis*, trigger secretion of pro-inflammatory cytokines (TNF-α and IL-6) in host immune cells [36]. These data suggest that DMWE alleviates periodontitis by inhibiting inflammatory factors induced by periodontal pathogens.

Periodontitis is a chronic multifactorial inflammatory disease leading to progressive destruction of the teeth-supporting apparatus, including the periodontal ligament and alveolar bone, caused by the accumulation of dental plaque [37,38]. Many pathogens are known to manipulate epithelial barriers and tight-junction (TJ) proteins, in order to enter host cells and tissues and resulting in increased bacterial invasion in periodontal lesions [39,40]. It has been demonstrated that IFN-γ, TNF- α, and IL-6, whose inflammatory cytokines impair TJ barrier function, are associated with a decrease in Zonula occludens-1 (ZO-1), occludin, and claudin-1 [37,41]. In this study, when DMWE was treated, it was confirmed that the inflammatory cytokines TNF- α and IL-6 were reduced. DMWE affected the cell junction, especially the TJ, by reducing inflammatory cytokines. Therefore, DMWE can be expected to alleviate periodontitis through the TJ mechanism.

Various indicators are used for the assessment of periodontitis. In the present study, micro-CT, histology, GI, and depth were evaluated. *P. gingivalis*, which is an oral bacteria, induced the infiltration of inflammatory cells and produced chemical mediators, such as inflammatory cytokines. We observed that the levels of GI and depth were increased in the DMWE-treated groups, which indicated that periodontitis had improved. Improvements in GI and depth alleviated problems, such as chewing disorders, caused by periodontitis, reducing discomfort during food intake, and body weight in the DMWE-treated group also increased (Table 4).

DMWE concentrations were established based on a previous study [42]. A reduction in alveolar bone loss was observed in all of the DMWE-treated groups compared with the PC. In particular, the BMD, BV, TV, and BV/TV ratios were markedly increased in DMWE-2 × MBC (Table 4). These results were observed by H&E staining (Figure 6). DMWE also exhibited significant effects at low doses compared with previous studies related to the effects of natural products for periodontitis treatment [43,44]. Thus, DMWE is highly effective at inhibiting alveolar bone loss and exhibits protective and preventive effects on periodontitis that occurs in periodontal tissue.

## 4. Materials and Methods

### 4.1. Sample Preparation

DMLs were provided by Hurim Hwangchil Co., Ltd. (Jinju, Korea), cultivated in Hadong-gun, Gyeongsangnam-do, Korea and identified by the National Institute of Biological Resource (NIBR). A voucher specimen of the plant (NIBRVP 0000823897) was also deposited at NIBR. The major components, chlorogenic acid and rutin, were purchased from Sigma-Aldrich (Saint Louis, MO, USA). Furthermore, 2,4,6-azino-bis(3-ethylbenzthiazoline-6-sulphnoic acid) (ABTS) and 2,2-diphenyl-1-picrydrazyl (DPPH) were purchased from Sigma-Aldrich along with the reagents used to measure anti-oxidant activity. Reagents and organic solvents, including methanol, acetonitrile, formic acid, water, and acetic acid, were purchased from J.T. Baker (Phillipsburg, NJ, USA) Other reagents were purchased and used as grade 1 reagents for analysis as indicated.

### 4.2. Preparation of DML Leaf Extracts

Extracts were prepared from DMLs as previously described [12]. Briefly, after washing and drying fresh DMLs, these were extracted using hot-water. The extracted solution was filtered, concentrated using an evaporator under vacuum, and lyophilized. The extract was stored at −20 °C until used in the assays. DML hot-water extracts were named DMWE.

### 4.3. Conditions of HPLC Analysis for Chlorogenic Acid and Rutin

Chlorogenic acid and rutin analysis was done by high-performance liquid chromatography (HPLC, Agilent 1200 system, Boeblingen, Germany) on a reversed-phase Eclipse Plus C_18_ column (4.6 × 250 mm, 5μm; Agilent, Santa Clara, CA, USA) at a column temperature of 30 °C with a flow rate of 1.0 mL/min. The mobile phase (A) was 0.1% formic acid in water (*v*/*v*) and the mobile phase (B) was 0.1% formic acid in acetonitrile (*v*/*v*). Mobile phase A was maintained at 100% at 0 min and mobile phase B increased slowly to 100% over 56 min. The analysis was performed at a wavelength of 310 nm and 20 μL was injected into the instrument. For each tested compound, a calibration curve was obtained by plotting the peak areas versus the standard amounts. The content of the compounds in the samples was calculated using the regression parameters of the calibration curves. Chlorogenic acid: y = 52,007x − 239.10 (R^2^ = 0.9998), Rutin: y = 10,820x − 265.26 (R^2^ = 0.9979).

### 4.4. Determination of Total Polyphenol and Flavonoid Content

Total polyphenol content was quantitated, following a previously published protocol [12]. Briefly, 1 mL of the DML extracts was diluted with 9 mL water and oxidized with 1 mL of diluted Folin-Ciocalteu reagent (Sigma-Aldrich) for 8 min at room temperature followed by neutralization with 4 mL sodium carbonate solution (7.5%, *w*/*v*). The mixture was diluted with water to a final volume of 16 mL and incubated for 2 h at room temperature. The absorbance at 725 nm was measured with a UV-VIS spectrophotometer (Libra S 35, Biochrom, Cambridge, England, UK). A blank sample of water in place of DMWE was used to distinguish background absorbance. The total polyphenol acid and flavonoid content were determined from calibration curves using gallic acid and quercetin acid.

### 4.5. DPPH Free Radical Scavenging Assay

The anti-oxidant capacity of the DMWE was evaluated using the DPPH free radical scavenging assay. The DPPH radical scavenging assay was done as described previously [45] using vitamin C as a positive control [46]. DPPH (100 µL amounts of a 0.2 mM solution in methanol) was mixed with 100 µL amounts of ascorbic acid solution or DMWE and the mixtures were incubated in the dark for 30 min at room temperature. The absorbance at 518 nm was measured using a microplate reader (Mobi, MicroDigital Co., Seoul, Republic of Korea). The DPPH radical scavenging effect was calculated as a percentage using the following equation:

$$\mathrm{DPPH}\;\mathrm{radical}\;\mathrm{scavenging}\;\mathrm{activity}\;(\%)=(\frac{A_{\mathrm{Contorl}}-A_{\mathrm{Sample}}\;}{A_{\mathrm{Contorl}}})\times 100$$

where A_Contorl_ is the absorbance of the control incubated with methanol and DPPH working solution (1 mL), and A_Sample_ is the absorbance of the samples. Anti-oxidant activity was calculated based on IC_50_ (μg/mL) values from the data of the DPPH free radical scavenging activities of the sample [47].

### 4.6. ABTS Radical Scavenging Activity Assay

ABTS radical scavenging activity was determined following a previous study. ABTS (Sigma-Aldrich, St. Louis, MO, USA) and potassium persulfate (Sigma-Aldrich, St. Louis, MO, USA) were dissolved in distilled water. ABTS solution 7 mM was mixed 1:1 (*v*/*v*) with potassium persulfate solution. Then, 2.4 mM was incubated in the dark for 16 h and the absorbance at 734 nm was measured and adjusted to 1.0 using distilled water. Ten microliters of 1, 5, 10, and 50 mg/mL of DMWE were mixed with 190 μL of ABTS solution in a 96-well plate and incubated for 10 min at room temperature and the absorbance at 734 nm was read using a microplate reader. Normal and positive controls were added to diluted water and vitamin C (1.0 mg/mL) instead of DMWE. The ABTS radical scavenging effect was calculated as a percentage using the following equation:

$$\mathrm{ABTS}\;\mathrm{radical}\;\mathrm{scavenging}\;\mathrm{activity}\;(\%)=(\frac{A_{\mathrm{Contorl}}-A_{\mathrm{Sample}}\;}{A_{\mathrm{Contorl}}})\times 100$$

A_Contorl_ is the absorbance of the control incubated with methanol and ABTS working solution (1 mL), and A_Sample_ is the absorbance of the samples.

### 4.7. NO Assay

RAW 264.7 cells were obtained from the Korea Cell Link Bank and cultured in Dulbecco’s Modified Eagle’s Medium containing 10% fetal bovine serum (Thermo Fisher Scientific, Inc., Waltham, MA, USA), 100 units/mL penicillin, and were incubated for 24 h at 37 °C in a CO_2_ incubator. RAW 264.7 cells were seeded into 24-well plates at a density of 5 × 10 ^5^ cells/well and incubated for 12 h. Next, cells were treated with 1, 10, 50, and 100 μg/mL of DMWE and incubated for 30 min. After various treatments, cell culture medium was mixed 1:1 (*v*/*v*) with Griess reagent (Sigma-Aldrich, St. Louis, MO, USA) and the NO concentration was measured at 540 nm using sodium nitrite (NaNO_2_) as a standard.

### 4.8. PGE_2_ Production Inhibition Activity

RAW 264.7 cells were seeded into 96-well microplates at 1.5 × 10 ^5^ cells/well and incubated at 37 °C in 5% CO_2_ for 6 h. Next, Raw 264.7 cells were pre-treated with 1, 10, 50, and 100 μg/mL of DMWE and incubated for 30 min followed by 1 μg/mL of LPS for 18 h. After incubation, PGE_2_ production in the medium was measured using a mouse enzyme-linked immunosorbent assay (ELISA) PGE_2_ kit (R&D Systems Inc., Minneapolis, MN, USA).

### 4.9. Animal

Twenty specific pathogen free (SPF) male Sprague–Dawley (SD) rats (255.2 ± 21.5 g, 7-week-old) were purchased from Samtako, Inc. (Osan, Republic of Korea). All rats were maintained at 23 ± 1.0 °C with 50 ± 10% humidity and a 12 h light–dark cycle. An air ventilation system was automatically controlled using an experimental animal breeding device (Three Shine, Daejeon, Republic of Korea). Food and drinking water were provided to the animals ad libitum and were autoclaved before use to maintain SPF conditions. The experiments began one week following adaptation. All animal experimental processes were approved by the Institutional Animal Care and Use Committee (IACUC) of Gyeongsang National University, under approval number GNU-221014-R0136, and performed in compliance with the guidelines of the IACUC of Gyeongsang National University, Republic of Korea.

### 4.10. Animal Preparation and Administration

In this study, the DMWE concentration was determined based on the data of the pre-vious antibacterial experiment [42]. The animals were randomly divided into 4 groups with 5 rats in each group. The non-ligated tooth, which was located on opposite of ligated-induced group (PC) was considered a normal control group (NC). To induce periodontitis, a 4–0 black silk suture (AILEE, Busan, Republic of Korea) was used for ligation for 2 days. Then, for 3 weeks, DMWE treated in periodontitis-induced rats was slowly absorbed after being kept in the mouth for a while. Experimental animal groups were set as follows; group 1, non-ligature (NC); group 2, ligature only (PC); group 3, treated minimum inhibitory concentration 3.13 mg/mL (DMWE-MIC); group 4, treated minimum bactericidal concentration 6.25 mg/mL (DMWE-MBC); and group 5, treated 2 × minimum bactericidal concentration 12.5 mg/mL (DMWE-2 × MBC).

**Figure 7:**
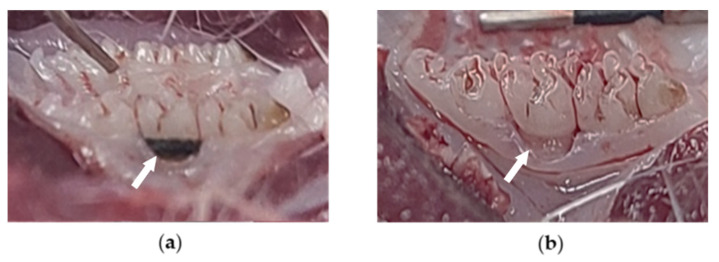
Ligation around the cervical portion of the maxillary second molar of rats with surgical black silk suture (4–0) to (**a**) cause periodontitis and (**b**) observe periodontitis-induced appearance after 3 weeks of ligation.

In a previous study, DMWE-MIC was used at 3.13 mg/mL and DMWE-MBC was 6.25 mg/mL, and the maximum dose that could be administered to the oral cavity of the rats was set at 0.1 mL through repeated tests. The process of inducing ligation in rats was done following the previous protocol [48]. Experimental rats were anesthetized with inhaled anesthetics (Surgivet, Waukesha, WI, USA) using a ventilation system (Model 687, Harvard Apparatus, Cambridge, UK) with 70% N_2_O, 2∼3% isoflurane (Hana Pharm Co., Ltd., Hwaseong, Republic of Korea), and 28.5% O_2_ gas. While maintaining anesthesia, the surrounding maxillary cervical second molars were ligated with 4–0 a black silk suture (Figure 7a) and periodontitis appeared after 3 weeks of ligation (Figure 7b). Body weight was measured before and after dental cervical ligation at 7, 14, and 21 days using scale electronic devices (Precisa Instrument, Dietikon, Switzerland).

### 4.11. Measurement of GI and Depth of Gingival Pocket

The animals were checked for ligation status, gingival bleeding, and degree of erosion each week after periodontal disease, according to the following criteria; Score 0, normal gingiva; Score 1, mild inflammation, slight edema, minor change in color, and absence of bleeding on probing; Score 2, moderate inflammation, edema, glazing, redness, and bleeding on probing; and Score 3, severe inflammation, extreme redness, presence of ulcers, edema, and severe bleeding. A periodontal probe (PCP UNC-15, HuFriedy^®^, Chicago, IL, USA) was inserted into the ligated tooth and the opposite normal tooth in the apical direction until tissue resistance was felt, and then the depth from the gingival margin to the base of the periodontal pocket was measured. The depth of the gingival pocket was measured as a value excluding the depth of the gingival pocket of the opposite normal tooth from the depth of the gingival pocket of the ligated tooth.

### 4.12. Collected Serum and Maxilla

At the end of the experiment, the animals were anesthetized with Zoletil^®^ (20 mg/kg; Virbac, Carros, France) and blood samples were collected from the heart. After euthanasia by cervical dislocation, the surrounding maxillary second molar, including the ligated tooth, was collected, fixed in 10% formaldehyde (Duksna Science, Seoul, Republic of Korea), and analyzed by micro-CT image. After centrifuging the blood samples collected from the rats, the serum was separated and inflammatory cytokines, including TNF-α and IL-6 levels, were measured using rat TNF-α and IL-6 ELISA kits (Sigma-Aldrich, St. Louis, MO, USA).

### 4.13. Micro-CT Imaging

**Figure 8:**
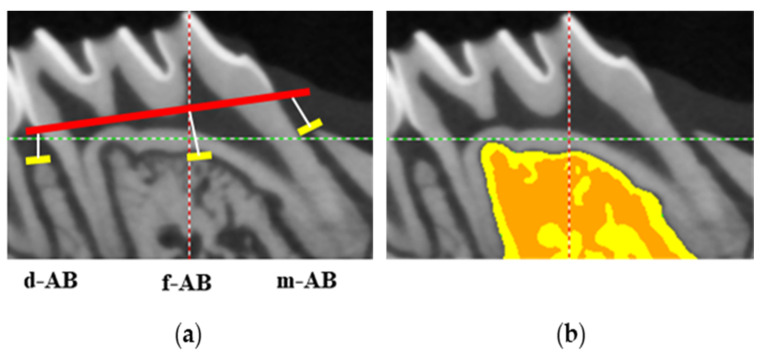
(**a**) Linear measurements of alveolar bone loss (AB) of the maxillary left second molars. The distance from the cementoenamel junction (CEJ) to the alveolar bone crest mesial to the second molar (m-AB), at the furcation of the second molar distal to the mesial root (f-AB) and distal to the second molar (d-AB) were measured. Red line means CEJ. (**b**) The region of the resultant 3D region of interest (ROI, yellow and deep yellow area) with the maxillary second molar. ROI is used to determine tissue volume (TV), bone volume (BV), and bone volume fraction (BV/TV) of the inter-radicular alveolar bone.

The maxillary specimens were scanned using a high-resolution micro-CT scanner (Quantum GX micro-CT Imaging System, PerkinElmer, Hopkinton, MA, USA). The tube was operated at a 90 kVp acceleration potential with a beam current of 180 μA for 3 min and an image pixel size of 19 μm. Reconstruction of images was analyzed with specific software. (OnDemand 3DTM, Cybermed, Seoul, Republic of Korea). Three-dimensional (3D) structures around the tooth and alveolar bone were evaluated at all widths. Periodontal bone heights were measured as the distances from the CEJ, ABC, and root surface. A line was drawn between the structures to be analyzed. The distance between the CEJ of the upper-left second molar and the coronal plane of the ABCs (CEJ-ABC) was measured at 3 sites per tooth for evaluation of alveolar bone resorption. In short, the distance from CEJ to the mesial alveolar bone (m-AB), the distance from the distal root bifurcation to the root mesial (f-AB), and the distance to the distal alveolar bone (d-AB) were measured (Figure 8a). In addition, based on a previous study [49], the inter-radical septum of the second molar, which is frequently used to measure the histomorphism of the trabecular bone, was selected as the region of interest (ROI) of the alveolar bone (Figure 8b). Next, the 3D microstructure of alveolar bone was analyzed from ROI using micro-CT software programs and the BMD, BV, TV, and BV/TV ratio were evaluated.

### 4.14. Statistical Analysis

Data are expressed as the mean ± SEM. Statistical comparisons were analyzed by a one-way analysis of variance and Duncan’s multiple range test using SPSS version 20 (IBM, New York, NY, USA). A *p*-value less than 0.05 was considered statistically significant.

## 5. Conclusions

This study demonstrates that DMWE decreased the cytokine production that is associated with the onset and recurrence of periodontitis. DMWE showed anti-inflammatory effects through a mechanism of up-regulated TJ. Therefore, DMWE has high anti-oxidant and anti-inflammatory effects, protecting periodontal tissue damage and tooth loss. Furthermore, it suggests that DMWE can be used for the prevention and treatment of chronic periodontitis caused by a continuously inflammatory process in acute periodontitis.

## Figures and Tables

**Table 1:** Total polyphenol and flavonoid contents in DMWE.

Items	Concentration
Total polyphenol (GAE mg/g) ^1^	55.32 ± 2.15
Total flavonoid (QE mg/g) ^2^	11.98 ± 0.12

^1^ Total polyphenol content is expressed as gallic acid equivalents (GAE); ^2^ Total flavonoid content is expressed as quercetin equivalent (QE).

**Table 2:** Body weight in periodontitis-induced rats following DMWE treatment.

Groups	Body Weight (g) after Treatment of DMWE (Weeks)				Weight Gain (g)
	0 *	1	2	3	
PC	269.1 ± 7.8	294.0 ± 14.9	307.4 ± 15.6	308.3 ± 15.2	39.9 ± 10.7 ^a^
DMWE-MIC	266.1 ± 6.5	298.4 ± 13.5	307.6 ± 12.5	309.0 ± 13.9	40.5 ± 8.7 ^a,b^
DMWE-MBC	271.0 ± 9.3	304.2 ± 16.9	317.4 ± 18.6	320.2 ± 16.2	49.2 ± 8.1 ^a,b^
DMWE-2 × MBC	261.9 ± 5.8	298.2 ± 7.0	311.8 ± 8.4	315.6 ± 11.5	53.7 ± 6.5 ^b^

* Before inducing periodontitis. PC, positive control treated with distilled water after inducing periodontitis; DMWE-MIC administered with 0.1 mL MIC (3.13 mg/mL) of DMWE; DMWE-MBC administered with 0.1 mL MBC (6.25 mg/mL) of DMWE; DMWE-2 × rMBC administered with 0.1 mL 2 × MBC (12.5 mg/mL) of DMWE. ^a,b^ Mean values with different superscript letters in the same column indicate statistical significance at *p* < 0.05 by one-way ANOVA.

**Table 3:** Serum TNF-α and IL-6 concentrations after DMWE treatment in rats with induced periodontitis for 3 weeks.

Group	TNF-α (pg/mL)	IL-6 (pg/mL)
PC	0.098 ± 0.008 ^a^	0.089 ± 0.010 ^a^
DMWE-MIC	0.090 ± 0.006 ^a,b^	0.081 ± 0.021 ^a,b^
DMWE-MBC	0.086 ± 0.011 ^b,c^	0.075 ± 0.012 ^a,b^
DMWE-2 × MBC	0.081 ± 0.007 ^c^	0.071 ± 0.010 ^b^

PC, positive control treated with distilled water after inducing periodontitis; DMWE-MIC administered with 0.1 mL MIC (3.13 mg/mL) of DMWE; DMWE-MBC administered with 0.1 mL MBC (6.25 mg/mL) of DMWE; DMWE-2 × MBC administered with 0.1 mL 2 × MBC (12.5 mg/mL) of DMWE. ^a,b,c^ Mean values with different superscript letters in the same column indicate statistical significance at *p* < 0.05 by one-way ANOVA.

**Table 4:** Gingival index and depth of the gingival pocket after administration of DMWE in rats with induced periodontitis for 3 weeks.

Group	Gingival Index	Gingival Sulcus Depth (mm)
NC	0.00 ± 0.00 ^a^	0.00 ± 0.00 ^a^
PC	1.20 ± 0.27 ^b^	1.38 ± 0.16 ^b^
DMWE-MIC	1.00 ± 0.00 ^b,c^	1.14 ± 0.35 ^b,c^
DMWE-MBC	0.80 ± 0.45 ^b,c^	1.08 ± 0.29 ^b,c^
DMWE-2 × MBC	0.60 ± 0.55 ^a,c^	0.94 ± 0.26 ^c^

NC, normal control group composed of opposite molars that did not induce periodontitis; PC, positive control treated with distilled water after inducing periodontitis; DMWE-MIC administered with 0.1 mL MIC (3.13 mg/mL) of DMWE; DMWE-MBC administered with 0.1 mL MBC (6.25 mg/mL) of DMWE; DMWE-2 × MBC administered with 0.1 mL 2 × MBC (12.5 mg/mL) of DMWE. ^a,b,c^ Mean values with different superscript letters in the same column indicate statistical significance at *p* < 0.05 by one-way ANOVA.

**Table 5:** Bone mineral density and bone morphological microstructure of the alveolar bone in rats with induced periodontitis after DMWE administration for 3 weeks.

Group	BMD (mg/cm^3^)	BV (mm^3^)	TV (mm^3^)	BV/TV (%)
PC	924.1 ± 39.3 ^a^	0.75 ± 0.08 ^a^	1.29 ± 0.09 ^a^	58.08 ± 2.45 ^a^
DMWE-MIC	931.4 ± 42.5 ^a,b^	0.78 ± 0.05 ^a,b^	1.33 ± 0.08 ^a,b^	58.33 ± 1.29 ^a,b^
DMWE-MBC	942.8 ± 24.4 ^a,b^	0.81 ± 0.09 ^a,b^	1.36 ± 0.10 ^a,b^	59.44 ± 3.41 ^a,b^
DMWE-2 × MBC	954.4 ± 36.7 ^b^	0.86 ± 0.08 ^b^	1.40 ± 0.06 ^b^	61.04 ± 4.29 ^b^

BMD, bone mineral density; BV, bone volume; TV, tissue volume. PC, positive control treated with distilled water after inducing periodontitis; DMWE-MIC administered with 0.1 mL MIC (3.13 mg/mL) of DML hot-water extracts (DMWE); DMWE-MBC administered with 0.1 mL MBC (6.25 mg/mL) of DMWE; DMWE-2 × MBC administered with 0.1 mL 2 × MBC (12.5 mg/mL) of DMWE. ^a,b^ Mean values with different superscript letters in the same column indicate statistical significance at *p* < 0.05 by one-way ANOVA.

## Data Availability

The data presented in this study are available upon request from the corresponding author.

## Sample Availability

Samples of the compounds are not available from the authors.
